# MERTK-related engulfment of norepinephrinergic neuronal presynaptic membrane by astrocytes following acute TBI and potentiation effect of peripheral nerve modulation

**DOI:** 10.1080/07853890.2026.2735720

**Published:** 2026-09-27

**Authors:** Xixian Liao, Ming Li, Fei Niu, Zilong Zhao, Jun Hong, Yu Deng, Yu Yan, Chuanhang He, Yanhao Liao, Ruilin Li, Yuan Zhuang, Guoyi Gao

**Affiliations:** ^a^ Department of Neurosurgery, Beijing Tiantan Hospital, Capital Medical University, Beijing, China; ^b^ Beijing Key Laboratory of Central Nervous System Injury, Beijing Neurosurgical Institute, Capital Medical University, Beijing, China; ^c^ Tianjin Institute of Neurology, Department of Neurosurgery, Tianjin Medical University General Hospital, Tianjin, China; ^d^ Department of Neurosurgery, Tangshan Gongren Hospital, Hebei Medical University, Tangshan, China

**Keywords:** Astrocytes, locus coeruleus, phagocytosis, right median nerve stimulation, traumatic brain injury

## Abstract

**Objectives:**

The study investigated the locus coeruleus (LC) changes after acute traumatic brain injury (TBI) and the effects of peripheral nerve modulation (PNM) in a mouse model.

**Methods:**

We performed closed controlled impact to simulate TBI in mice. We adopted electrical stimulation of the right median nerve as a method of PNM. Low-frequency and high-frequency peripheral nerve modulation (LF-PNM and HF-PNM) were administered on the 1st, 2nd, and 3rd days after the injury. We conducted a cell interaction analysis on the single-cell data of the mouse brainstem from the previous stage. Meanwhile, we also performed arousal and stress behavioral tests, immunofluorescence staining for apoptosis and activation of LC norepinephrinergic neurons, and examination of astrocytic phagocytosis in association with MERTK expression.

**Results:**

Acute TBI induced apoptosis of LC norepinephrinergic neurons alongside behavioral impairments. TBI triggered reactive astrogliosis in the LC, with elevated GFAP-Lamp2 colocalization. Increased GFAP-NET colocalization indicated astrocytic engulfment of presynaptic membrane proteins from norepinephrinergic neurons. Cell interaction analysis identified MERTK-mediated astrocyte-norepinephrinergic neuron crosstalk; GFAP-MERTK immunofluorescence confirmed spatially increased astrocytic MERTK expression linked to NET phagocytosis. LF-PNM further enhanced astrocytic phagocytosis of LC norepinephrinergic presynaptic proteins after TBI, reduced neuronal apoptosis, facilitated neuronal activation, and rescued arousal and stress functions.

**Conclusion:**

After acute TBI, LC astrocytes phagocytose norepinephrinergic presynaptic proteins *via* MERTK. LF-PNM enhances this process, improving post-TBI arousal and stress dysfunction.

## Key messages

1. Acute TBI induces extensive apoptosis of LC norepinephrinergic neurons and robust reactive activation of astrocytes, accompanied by markedly elevated astrocytic phagocytosis of norepinephrinergic presynaptic membranes via the MERTK-dependent pathway.
2. PNM exerts differential effects on LC norepinephrinergic neurons and astrocytes after acute TBI: LF-PNM suppresses neuronal apoptosis and further boosts astrocyte reactive activation and phagocytic activity, whereas HF-PNM hardly affects astrocyte activation yet impairs their phagocytic capacity.
3. Acute TBI causes deficits in arousal and stress functions, and PNM ameliorates these neurological impairments, with LF-PNM showing superior therapeutic efficacy.

## Graphical abstract

**Figure:**
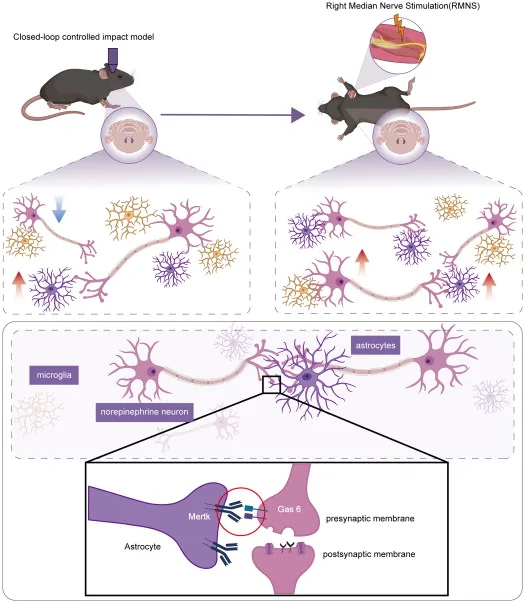

## 1. Introduction

Traumatic brain injury (TBI) has been an important subject in medical research for a long time. After the first impact, other injuries continue to occur and affect many neural networks in the whole brain. These slow and complicated processes lead to abnormal brain function, behavioral changes and long-term arousal problems [1,2]. Therefore, understanding the pathological processes related to TBI is crucial for improving neurotherapeutic approaches.

The brainstem, that vital hub linking the cerebral cortex and the spinal cord, plays a crucial role in managing the autonomic nervous system and orchestrating various essential bodily functions, including breathing [3,4], heart rate regulation [5,6], and processing sensory information from the upper body. These sensory inputs ultimately converge within the reticular activating network, a component crucial for modulating the state of alertness [7,8], levels of attention, and influencing conscious activity [9,10]. Among the various nuclei in the brainstem, the locus coeruleus (LC) plays a crucial role in maintaining the arousal and stress functions and is vulnerable to TBI [11]. Philipp O. Valko et al. have demonstrated that the number of norepinephrinergic neurons in the LC decreases following TBI [12]. After TBI, programmed cell death and inflammatory responses are triggered, accompanied by the reorganization of neural connections within the LC. Collectively, these findings suggest that the LC may serve as a critical target for the treatment of arousal disorders associated with TBI.

Astrocytes are a typical type of neuroglial cell whose end feet ensheathe synapses, thereby forming the tri-partite synapse and playing a crucial role in synaptic transmission [13,14]. Recent studies have shown that astrocytes express a range of phagocytosis-related genes, which means that astrocytes have phagocytic capabilities [15,16]. By swallowing and eliminating synapses, stellate phagocytosis helps to protect synaptic networks and their ability to change [17]. MERTK, a member of the MER/AXL/TYRO3 kinase receptor family, is expressed in glial cells and endothelial cells, with predominant expression in glial cells [18,19]. Previous studies have demonstrated that MERTK cooperates with the integrin pathway to regulate the CrKII/DOCK180/Rac1 module, which controls the rearrangement of the actin cytoskeleton during phagocytosis [20,21]. Studies by Chung and his colleagues show that astrocytes use Megf10 and Mertk mechanisms to eliminate synapses, which is observed in both developing and fully mature brains [22]. Current researches related to astrocytes phagocytosis of synapses have focused on the hippocampus [23] and cortex [24], while it remains unclear whether astrocytes phagocytose synapses in the LC after TBI.

Numerous studies have shown that peripheral nerve modulation (PNM) can cause histological changes in the central nervous system [25,26]. According to research conducted by Lv and colleagues, transcutaneous auricular vagus nerve stimulation (taVNS) in MRL/lpr mice produced beneficial effects throughout the peripheral and central nervous systems by stimulating tyrosine hydroxylase-positive neurons within the locus coeruleus [27]. Ming Tatt Lee and his team showed that stimulating the median nerve in mice with TBI could turn on the orexin neurons in the lateral hypothalamus (LH). This activation led to the release of orexin into the ventrolateral periaqueductal gray (vIPAG), a part of the midbrain that helps to suppress pain signals, effectively reducing neuropathic pain in the animals [28]. The effects of PNM on the phagocyte of astrocytes within the LC following acute TBI remain ambiguous and vague. In this study, we explored the phagocyte of astrocytes within the LC following acute TBI, as well as the effects of PNM on the phagocyte of astrocytes, and investigated its underlying mechanisms.

## 2. Materials and methods

### 2.1. Animals

Female C57BL/6 mice, weighing in at 20-25 grams apiece, were acquired from the Beijing Vital River Laboratory Animal Technology Co., Ltd. These animals are free from specific pathogens and were chosen for a study involving 60 mice, with 15 in each group. This study was approved by the Laboratory Animal Welfare and Ethics Committee of the Beijing Neurosurgical Institute (approval number, BNI202412005), and all the procedures were in accordance with the International Committee’s Guide for the Care and Use of Laboratory Animals. The research followed the guidelines set by the *in vivo* experiment report for animal studies. The mice were housed in a state-of-the-art environment, with five to a cage, a 12-hour light/dark schedule, and temperatures maintained at 22 ± 2 °C. Humidity levels were kept between 50 and 60%, and the animals were given access to food and water at any time. Mice were acclimated for 1 week and stratified by body weight and randomly assigned to four groups using a computer-generated random number table with block randomization (block size = 4): control group (*n* = 15), TBI group (*n* = 15), TBI+low-frequency peripheral nerve modulation (LF-PNM) group (*n* = 15), and TBI+high-frequency peripheral nerve modulation (HF-PNM) group (*n* = 15). To prevent any bias, we employed sealed, opaque envelopes that were cracked open by an impartial investigator just before the modeling or treatment phase got underway. The entire experiment calls for a grand total of 60 mice to make it all work.

### 2.2. Control group

In this experiment, the control group received a blank control, meaning no external intervention was applied.

### 2.3. Closed controlled impact (CCI) model

The closed controlled impact model was applied to the mice in the TBI group to induce TBI. Animals fell asleep with 5% isoflurane (provided by RWD Life Sciences Co., Ltd.) in 100% oxygen at a rate of 1 L/min, and then continued anesthesia with 2% isoflurane (Sigma). The animals were fixed in a prone position, and the skull was exposed using a midline incision. Then the midpoints of the anterior and posterior fontanelles were located and marked. Using the electromagnetic device CCI (Hartrath instrument CCI (Pinpoint PCI3000 precision leather impactor), mice experienced a closed controlled impact. This is done with a flat tip with a diameter of 3.0 mm, adjusted to a speed of 3.0 m/s, a penetration depth of 2.5 mm and a contact duration of 50 milliseconds. When a small amount of bleeding was observed at the impact site (Figure 1A and B), the closed controlled impact model was considered to be successfully established. Finally, the skin was sutured using 4-0 nylon wire. We used the Modified Neurological Severity Score (mNSS) scale to assess the severity of neurological deficits in mice at 24 h post-TBI. As shown in Table 1, the evaluation includes tests of reflexes, balance, sensory and motor abilities. Lower scores indicate better neurological outcomes [29]. The mNSS of the mice in this model were stable, ranging from 6 to 8.

**Figure 1.**
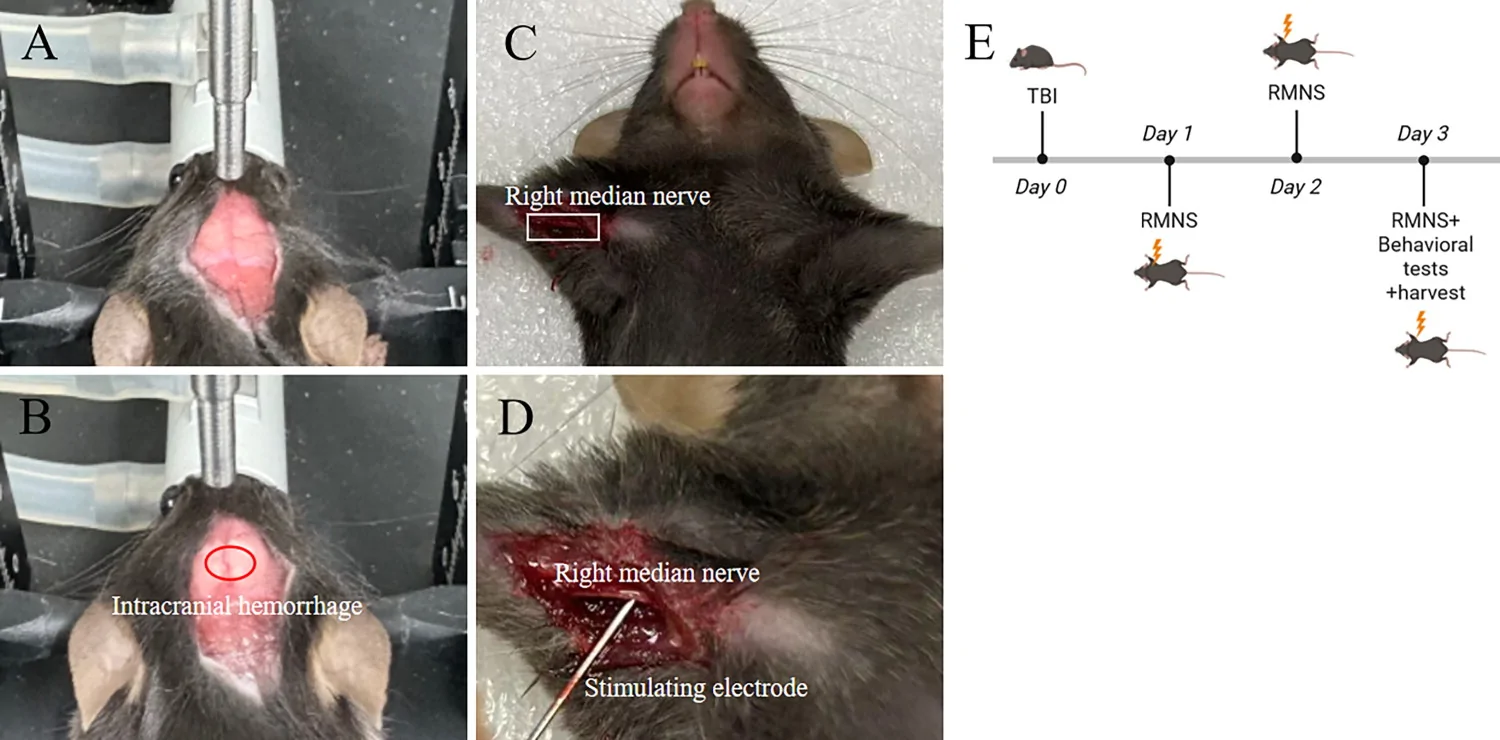
Closed controlled impact model and electrical stimulation of the right median nerve (RMNS). (A and B) Diagrams before and after TBI, respectively. (C) Anatomical diagram of the right median nerve. The right median nerve is within the white box. (D) Diagram displaying RMNS. (E) Timeline of RMNS.

**Table 1.** Modified neurological severity score (mNSS) scale.

Evaluation Indicator	Score
**1. Motor Function Assessment**	6
1.1 Tail Suspension Test	3
Forelimb flexion	1
Hindlimb flexion	1
Head deviation from vertical axis >10° within 30s	1
1.2 Free Walking on Flat Ground (normal = 0, max = 3)	3
Normal walking	0
Inability to walk in a straight line	1
Tilt/circling toward the hemiplegic side	2
Falling toward the hemiplegic side	3
**2. Sensory Function Assessment**	8
2.1 Placing Test (visual and tactile test)	1
2.2 Proprioception Test (deep sensation, limb muscle resistance test)	1
2.3 Beam Walking Test (normal = 0, max = 6)	6
Stable posture, can balance	0
Grasps one side of the beam	1
Hugs the beam, one limb falls off	2
Hugs the beam, two limbs fall off or rotates (>60s)	3
Attempts to balance but eventually falls (>40s)	4
Attempts to balance but eventually falls (>20s)	5
Falls, unable to attempt balance or hang on (<20s)	6
**3. Loss of Reflexes or Abnormal Movements**	4
3.1 Pinna Reflex (head shake upon light touch of the ear canal)	1
3.2 Corneal Reflex (eye blink upon light touch of the cornea with cotton)	1
3.3 Startle Reflex (limb movement caused by a brief noise from snapping paper)	1
3.4 Epilepsy, myoclonus, dystonia	1
Total Score	18

### 2.4. Parameters of PNM

The PNM was delivered with a digital neurophysiological system(Neuron-Spectrum-5, Neurosoft Llc.) and the parameters were based on previous research [30,31]. The method of PNM was electrical stimulation of the right median nerve. The PNM is divided into HF-PNM and LF-PNM: (1) HF-PNM:100μA, 40Hz, 10s ON and 20s OFF, total length of 20 min, with 10 ms pulse width; (2) LF-PNM:100μA, 2Hz, 10s ON and 20s OFF, total length of 20 min, with 10 ms pulse width. Figure 1C shows the anatomical location of the right median nerve in the mice. To ensure stable electrode placement on the mouse’s right median nerve for prolonged stimulation periods (Figure 1D), the animals were initially anesthetized with 5% isoflurane in an induction chamber before being maintained under 2% isoflurane *via* a nose-cone apparatus while resting on a heated table. Mice assigned to either the HF-PNM or LF-PNM groups were subjected to daily electrical stimulation either high-frequency or low-frequency for three consecutive days. The complete protocol for PNM is illustrated in Figure 1E.

### 2.5. Euthanize the mice and remove the brain tissue

On the 3rd day after electrical stimulation of the right median nerve following modeling, the mice were anesthetized with 5% isoflurane (RWD Life Sciences Co., Ltd.) in pure oxygen at a rate of 1 L/min. Then, they kept 2% isoflurane (Sigma) under anesthesia by inhalation. After confirming that the mice were in a state of deep anesthesia, they were fixed in a supine position on a dissection table. The skin of the chest and abdomen was disinfected with 75% alcohol. Tissue scissors were used to make an incision upward along the abdominal wall to expose the bilateral thoracic cavities and the heart. The heart was stabilized with toothed forceps in the left hand, while a needle attached to a syringe was inserted into the left ventricle with the right hand. Simultaneously, an ophthalmic scissor was used to create an opening at the right atrial appendage. Perfusion was performed with ice-cold Phosphate Buffered Saline (PBS) until the liver turned pale and clear fluid flowed out from the right atrial appendage. After saline perfusion, 20 ml of 4% paraformaldehyde was infused. Successful perfusion was indicated by the whitening of the lungs, liver, and intestines, as well as the upward curling of the tail. Following perfusion, tissue scissors were used to detach the mouse head at the craniocervical junction. The scalp was incised to expose the skull. Hemostatic forceps were used to remove the skull carefully, avoiding damage to the brainstem and other brain tissues. The entire brain, including the brainstem, was then removed. Furthermore, animals were monitored at least twice daily for signs of pain or distress. Humane endpoints included: (1) body weight loss exceeding 20% of baseline; (2) body condition score (BCS) ≤ 2 (on a 5-point scale) (Table 2); (3) inability to reach food or water for >24 h; (4) persistent hunched posture or lack of response to gentle stimuli. Any animal reaching one or more of these endpoints was immediately euthanized by carbon dioxide inhalation followed by cervical dislocation.

**Table 2.** Body condition score (BCS) scale.

BCS (5-point scale)	Description
5	Obese, spine not palpable
4	Good condition, spine palpable with fat covering
3	Moderate condition, vertebral segments distinguishable
2	Poor condition, spine prominently protruding, pelvic bones palpable
1	Cachectic, vertebral segments clearly visible, pelvic bones protruding, no fat

### 2.6. Behavioral tests

The researchers conducted all the behavioral tests without knowing the group assignments. Once the data was collected, it was unblinded, and a third researcher supervised the analysis.

#### 2.6.1. Righting reflex test

Three days after the controlled impact, four different groups were evaluated to evaluate their recovery reflex and see their ability to wake up. Mice were anesthetized with a mixture of 100% oxygen and 5% isoflurane (RWD Life Sciences Co., Ltd.), with a flow rate of 2 L/min for one minute and twenty seconds. When they were anesthetized, each mouse was placed in a cage and noticed the time it took them to fully recover to their normal position. The time required for mice in four groups to turn over was analyzed to evaluate their ability to wake up.

#### 2.6.2. Tail-flick latency analysis

The tail-flick test measures an instinctive response to a stimulus and is widely used to evaluate stress response function. In this procedure, we placed the mice into a cassette, leaving the tail outside, and approached it with a heat source. The tail-flick latency, which represents the time to tail-flick, was recorded and analyzed to compare the stress ability among the groups.

### 2.7. Immunofluorescence staining

The specific procedures for perfusion and brain tissue retrieval are detailed in section 2.5 of this part. After extraction, the brains of mice were preserved in 4% paraformaldehyde at 4 °C for 24 h. Then, the sample was dehydrated in PBS with 30% sugar solution. Coronal brain sections (10 µm in thickness) were prepared on a cryo-stat microtome (CM3050S, Leica) at −20 °C. These sections were washed in Tris-Buffered Saline with Tween-20 (TBST), treated with Phosphate Buffered Saline with Tween-20 (PBST) blocking solution containing 3% Fetal Bovine Serum (FBS) for one hour at room temperature, and finally treated with primary antibody diluted in 1%FBS at 4 °C. The primary antibodies used in this study were: Rabbit anti-Dopamine beta Hydroxylase (DBH) (ab209487, 1:500, Abcam), Rabbit anti-cFos (2250 T, 1:1000, Cell signaling Technology), Rabbit anti-Caspase-3 p12 (A5357, 1:500, Selleck), Mouse anti-Glial Fibrillary Acidic Protein (GFAP) (ab279290, 1:500, Abcam), Rabbit anti-Norepinephrine transporter (NET) (ab254361, 1:100, Abcam), Rabbit anti-Lysosome-associated membrane protein 2 (Lamp2) (bs-2739R, 1:200, Bioss), Rabbit anti-MERTK (ab184086, 1:500, Abcam). Tissues were incubated at 4 °C overnight, followed by incubation with thecorresponding secondary antibodies of Alexafluor 488 or 594 (1:500, Abcam) for 1 h at room temperature. Samples were mounted using Vectashield containing 4′,6-diamidino-2-phenylindole (DAPI) for cell nuclei staining (ab104139, Abcam).

### 2.8. Inference cell–cell interactions

The procedure for single-nucleus RNA-sequencing of brainstem tissues from control and acute TBI mice has been described in our previous study [32]. We use CellChat version 2.1.2 to analyze the possible signal exchange between different neuronal and non-neuronal cell populations. CellChat Toolbox provides a set of established ligand-receptor pairs, including a set of different cell types and communication routes. This database covers various interaction mechanisms, including soluble factors of signal transduction, binding to extracellular matrix, direct cell-to-cell contact, and non-vascular media such as neurotransmitter pathways. Inferring the process of intercellular communication requires identifying highly expressed genes, evaluating the possibility of communication, and determining which cell interactions are statistically significant.

Briefly, we first imported normalized single cell gene expression matrix and annotated cell metadata into CellChat object, with predefined cell type labels separating neuronal and non-neuronal populations. We then filtered lowly expressed genes and calculated the average expression level of each ligand and receptor within each cell group. Next, we projected gene expression onto the built in ligand-receptor interaction database to construct a cell-cell communication probability matrix, which quantifies the likelihood of signal transmission from sender cell populations to receiver cell populations. We performed permutation testing to assess statistical significance of each predicted communication interaction; only interactions with adjusted P-values below the defined threshold were retained as credible cell-cell communications. After constructing the global communication network, we summarized outgoing and incoming signaling strengths for each cell population to identify dominant signal senders and receivers. We further decomposed the network to highlight individual signaling pathways and ligand-receptor pairs mediating neuronal to non-neuronal crosstalk. Finally, we visualized violin plot and pathway activity heatmaps to interpret major signaling routes between neuronal and non-neuronal cell types.

### 2.9. Statistical analysis

The number of DBH^+^ neurons, DBH^+^caspase3^+^ neurons, DBH^+^cFos^+^ neurons and GFAP^+^ cells were analysed using ImageJ software(version 1.6). The immunofluorescence colocalization analyses of GFAP and NET, GFAP and Lamp2 as well as GFAP and MERTK were also performed using ImageJ software(version 1.6). All experimental results are presented as the mean ± standard deviations (SD). First, check the data of this study to see if they are normal and properly distributed. Students’ T-test is used to determine the statistical significance between two groups when the data is normal. In order to compare several groups with normal data, we use single factor ANOVA, followed by post-hoc LSD test when the variance is equal, or Dunnett T3 post-hoc test when the variance is unequal. Abnormal data were compared between two groups by Wilcoxon test, and several groups were compared by Kruskal-Wallis test. SPSS software 21.0 was used for statistical processing, and significant results were considered when *p* < 0.05. Visualize the results with GraphPad Prism 9 software.

### 2.10. Strategies for reducing confounding factors

To reduce the risk of confounding variables, all mice were the same age. They live in the same conditions: five in each cage, 12 h of light/dark cycle, 22 ± 2 °C temperature, 50% to 60% humidity, and free access to food and water. They have seven days to get used to it before the experiment begins. Mice were randomly assigned to groups using block randomization stratified by body weight. All surgical procedures were performed by the same experienced investigator between 9:00 and 11:00 AM. Behavioral tests were conducted during the light phase (9:00 AM–2:00 PM) with the experimenter blinded to group allocation.

## 3. Results

### 3.1. Changes in the LC during the acute phase of TBI

Given that LC is a key brainstem area responsible for controlling arousal and stress responses, we studied how norepinephrinergic neurons (mainly the dominant group of neurons in the LC) were affected after mice suffered acute TBI. The results showed that compared with control mice, apoptosis of norepinephrinergic neurons in the LC was significantly increased after acute TBI (Figure 2).

**Figure 2.**
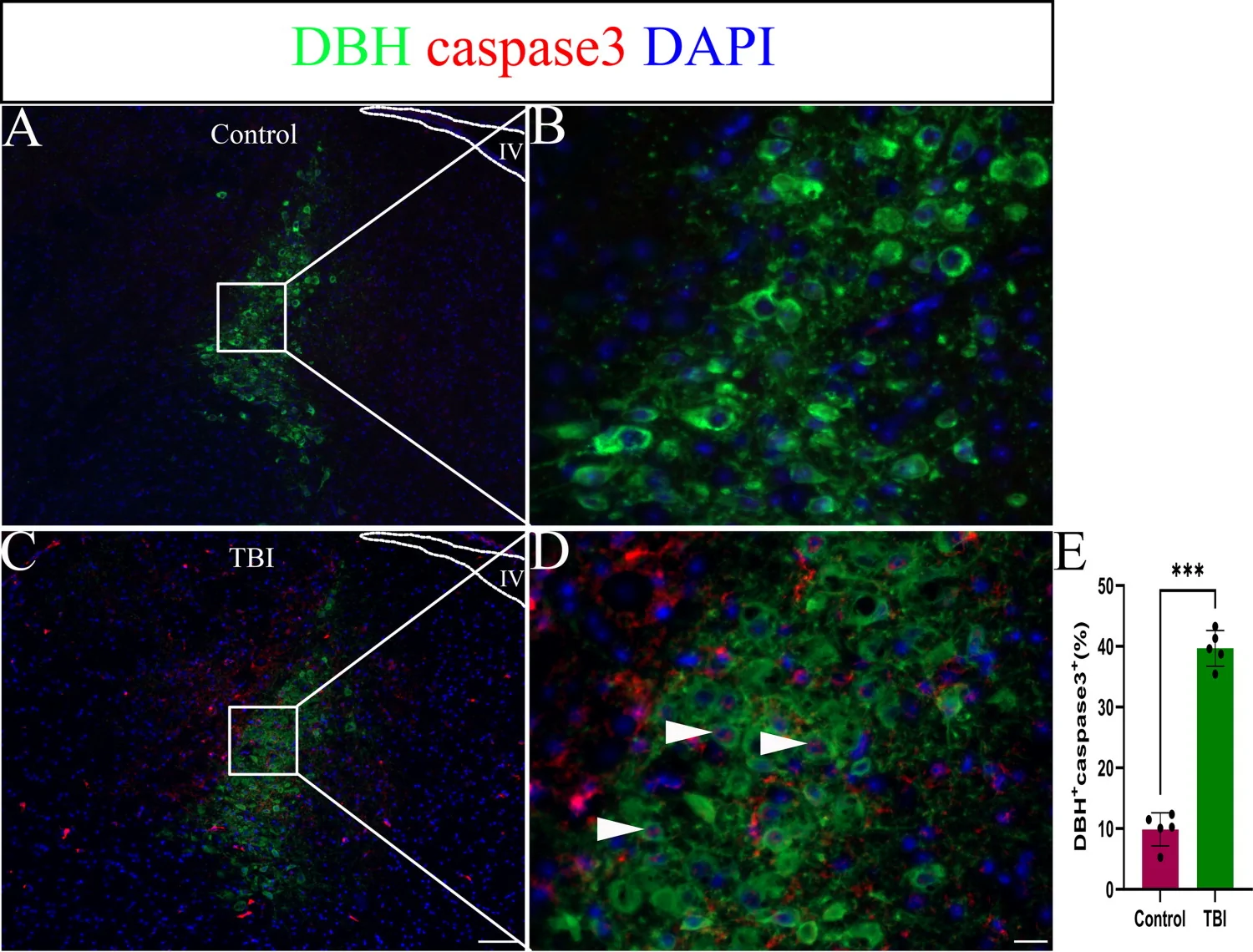
Acute TBI leads to increased apoptosis of norepinephrinergic neurons in LC. (A and B) Co-labeling of norepinephrinergic neurons and caspase3 in LC in control group. (C and D) Co-labeling of norepinephrinergic neurons and caspase3 in LC in TBI group. Co-labeled DBH and caspase3 in LC of the TBI group were shown by the head of white arrows in (D). (A and C) Sacle bar:100μm. (B and D) Scale bar: 20 μm. (E) Percentage of caspase3^+^ cells within DBH^+^ neurons in the LC of Control group mice and TBI group mice (each group, *n* = 5). t-tests employed for (E). (Contol group vs. TBI group, ***p < 0.001). IV:Fourth ventricle.

### 3.2. Robust engulfment in the LC during the acute phase of TBI

Numerous studies have reported that following TBI, glial cells at the site of injury phagocytose cellular debris and eliminate redundant synapses [33,34]. According to the results of Figure 2, we observed that following acute TBI, a large number of norepinephrinergic neurons in the LC underwent apoptosis, which may activate the phagocytic activity of glial cells, promoting their phagocytosis of cellular debris and redundant synapses.

In our previous study, we investigated the cell population distribution in the brainstem tissue of mice by performing single-cell sequencing combined with spatial transcriptomics analysis on the brainstem tissue [32]. Then, through Cellchat analysis, we found that in the brainstem, compared with microglia, astrocytes had α1-adrenergic receptors, which were more conducive to mutual recognition between astrocytes and norepinephrinergic neurons (Figure 3A). Therefore, we speculated that in the brainstem, astrocytes primarily exerted phagocytic actions on norepinephrinergic neurons to eliminate redundant synapses and remodel the relevant neural circuits.

**Figure 3.**
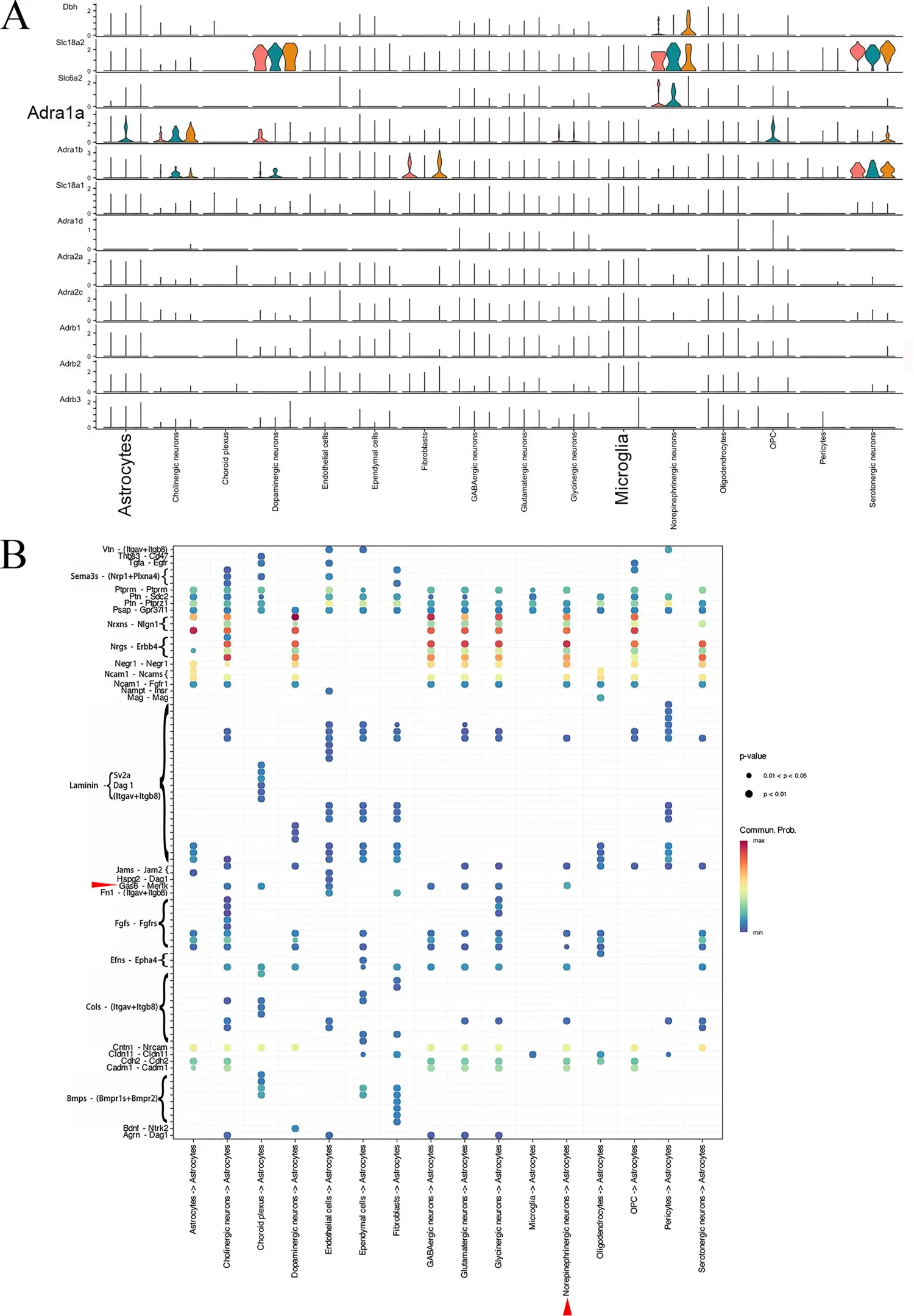
In the brainstem, compared to microglia, astrocytes are more likely to recognize norepinephrinergic neurons and exert phagocytic actions to clear redundant synapses. (A) Cellchat analysis showed that in the brainstem, compared with microglia, astrocytes have α1-adrenergic receptors. (B) Cellchat analysis showed that astrocytes and norepinephrinergic neurons are connected through ligands related to cell adhesion and phagocytosis.

To confirm the phagocytosis of astrocytes, we conducted a series of immunofluorescence experiments. During the acute phase of traumatic brain injury (TBI), astrocytes within the LC exhibit prominent reactive activation. Consistently, the expression level of glial fibrillary acidic protein (GFAP, an astrocyte-specific marker) is markedly elevated in the LC of TBI-injured mice relative to control animals, which supports this observation (Figure 4). In addition, the elevated co-location of GFAP and Lamp2 indicated robust phagocytosis of astrocytes (Figure 5). To visualize the elimination of norepinephrinergic synaptic terminals mediated by astrocytes, we co-labeled GFAP and NET (Norepinephrine transporter, a membrane transport protein located in the presynaptic membrane of norepinephrinergic neurons) with AlexaFluor 488 and 594 in the LC of mice in the control group and the TBI group. The results showed that colocalization of GFAP and NET was observed in the LC of both groups (Figure 6A–D). However, by calculating the fluorescence colocalization rate, we found that the colocalization of GFAP and NET in the LC was significantly increased after acute TBI (Figure 6E), indicating that astrocytes in the LC extensively phagocytose the presynaptic membranes of norepinephrinergic neurons during the acute phase of TBI. The Cellchat analysis also showed that astrocytes and norepinephrinergic neurons were connected through ligands related to cell adhesion and phagocytosis, which suggested that astrocytes played a phagocytic role in norepinephrinergic neurons (Figure 3B).

**Figure 4.**
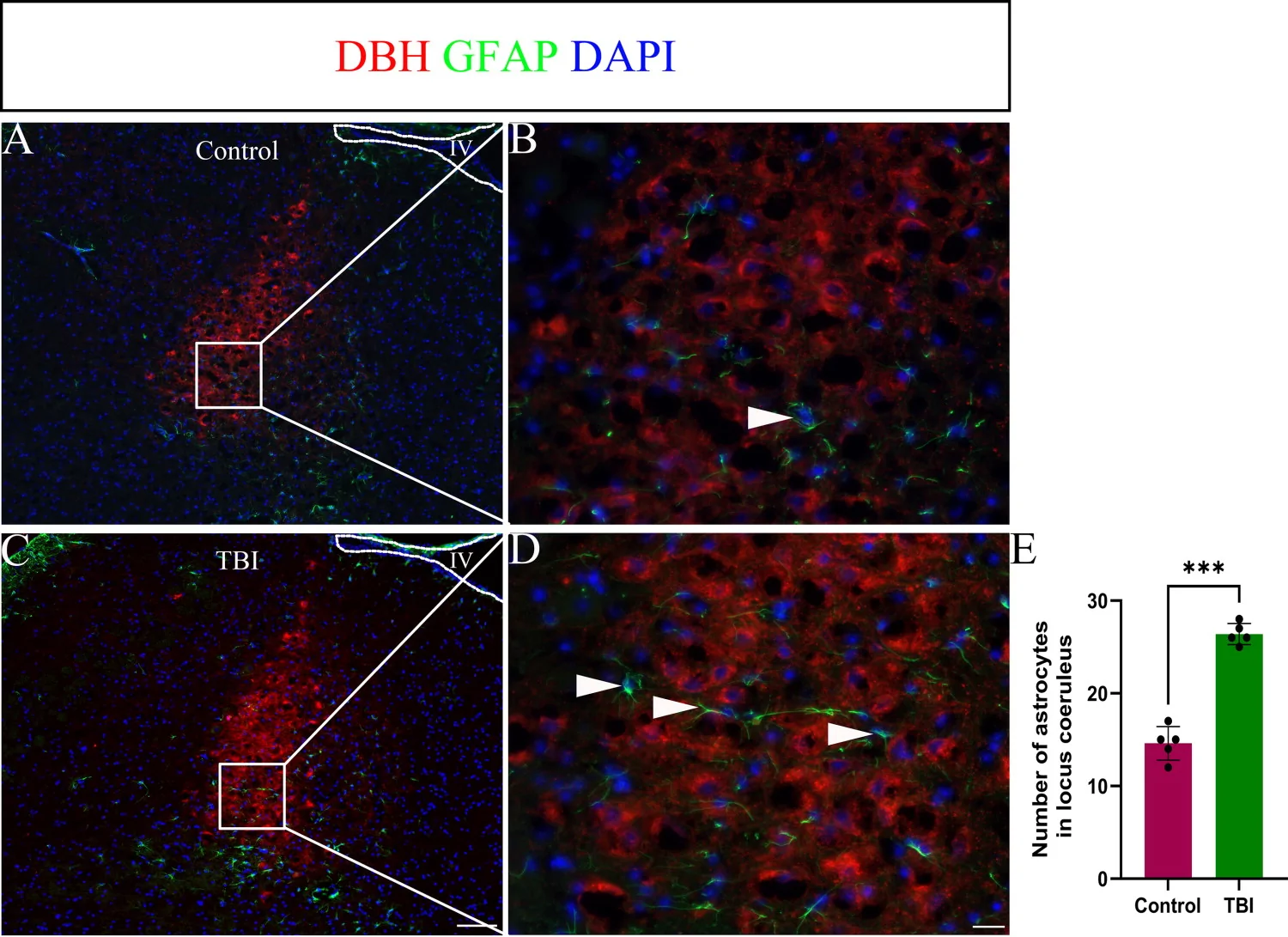
Reactive activation of astrocytes in LC following acute TBI. (A and B) GFAP expression level in LC of the control group. GFAP in LC of the control group were shown by the head of white arrows in (B). (C and D) GFAP expression level in LC of the TBI group. GFAP in LC of the TBI group were shown by the head of white arrows in (D). (A and C) Sacle bar:100μm. (B and D) Scale bar: 20 μm. (E) The number of astrocytes in LC of Control group mice and TBI group mice(each group, *n* = 5). t-tests employed for (E). (Contol group vs. TBI group, ***p < 0.001). IV:Fourth ventricle.

**Figure 5.**
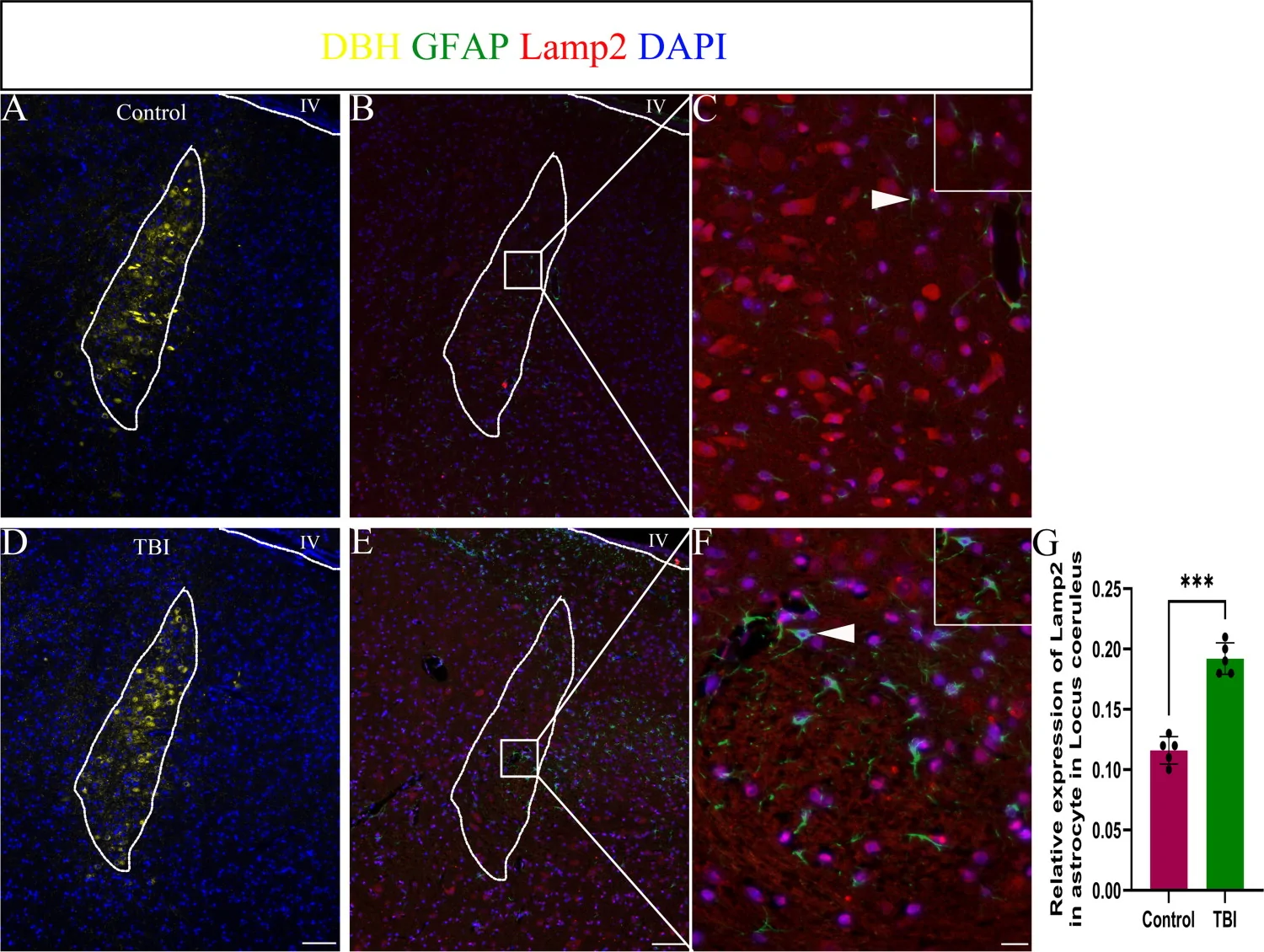
Astrocytes showed activated phagocytic ability in LC following acute TBI. (A and D) The location of LC in control group and TBI group. The irregular dashed line outlining the range of DBH represents the LC. Scale bar: 100 μm. (B and C) Co-labeling of astrocytes and Lamp2 in LC of control group. Co-labeling of GFAP and Lamp2 in LC of control group were shown by the head of white arrows in (C), with the inset in the upper right corner showing an enlarged view of the indicated area. (E and F) Co-labeling of astrocytes and Lamp2 in LC of TBI group. Co-labeling of GFAP and Lamp2 in LC of TBI group were shown by the head of white arrows in (F), with the inset in the upper right corner showing an enlarged view of the indicated area. (B and E) Scale bar: 100 μm. (C and F)Scale bar: 20 μm. (G) Co-labeling of Lamp2 and GFAP in LC is enhanced following TBI, which indicates an increased phagocytic capacity of astrocytes. (each group, *n* = 5). t-tests employed for (G). (Contol group vs. TBI group, ***p < 0.001). IV:Fourth ventricle.

**Figure 6.**
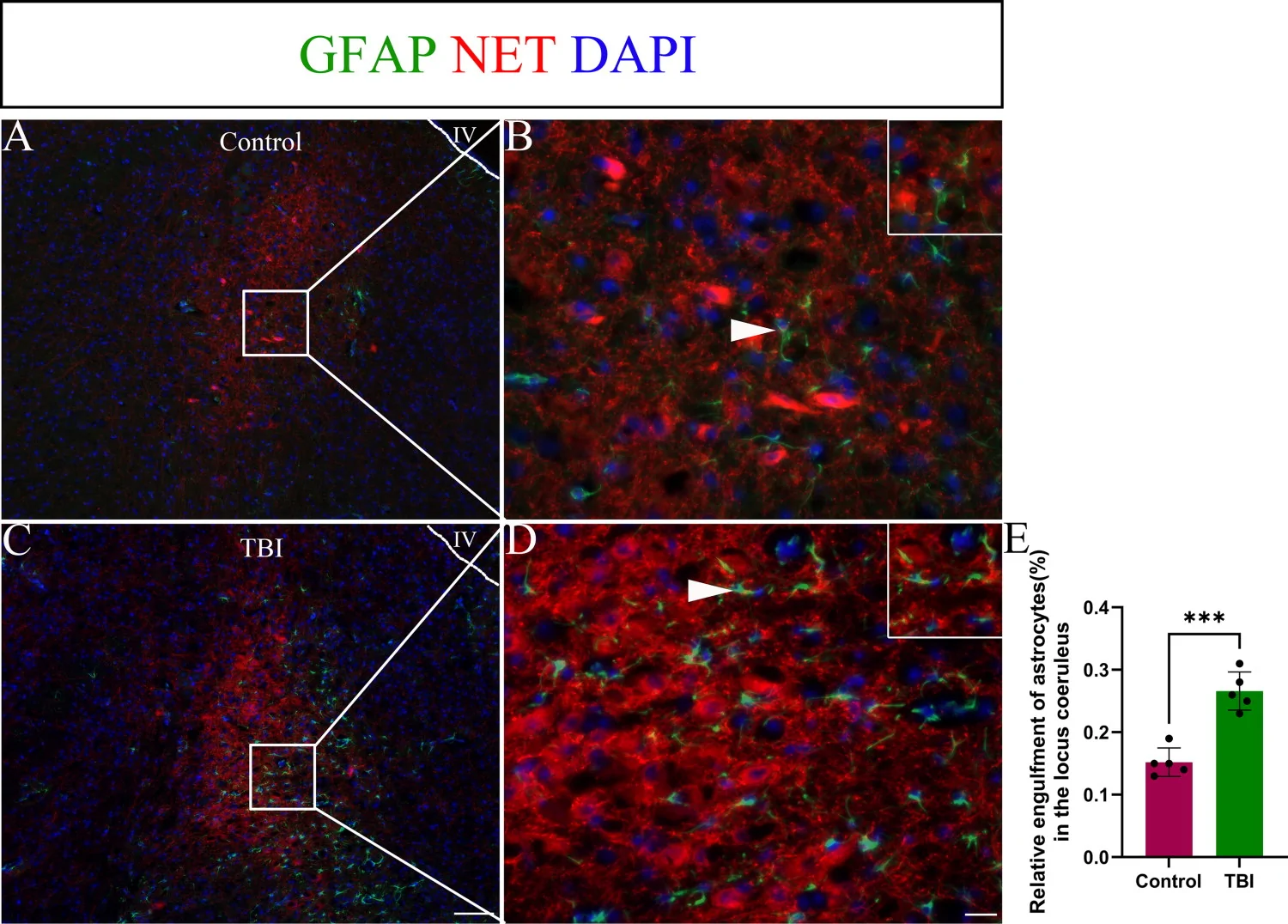
Astrocytes showed robust engulfment of NET in LC following acute TBI. (A and B) Co-labeling of astrocytes and NET in LC of control group. Co-labeling of GFAP and NET in LC of control group were shown by the head of white arrows in (B), with the inset in the upper right corner showing an enlarged view of the indicated area. (C and D) Co-labeling of astrocytes and NET in LC of TBI group. Co-labeling of GFAP and NET in LC of TBI group were shown by the head of white arrows in (D), with the inset in the upper right corner showing an enlarged view of the indicated area. (A and C) Sacle bar:100μm. (B and D) Scale bar: 20 μm.(E) The engulfment ability of NET in astrocytes was assessed by the co-labeled ratio of GFAP to NET. (each group, *n* = 5). t-tests employed for (E). (Contol group vs. TBI group, ***p < 0.001). IV:Fourth ventricle.

### 3.3. MERTK-related engulfment of NET by astrocytes during the acute phase of TBI

Figure 3B indicated that astrocytes in the brainstem region may mediate the phagocytosis of norepinephrinergic neuronal synapses through MERTK. To clarify during the acute phase of TBI, whether the phagocytosis of norepinephrinergic synaptic terminals by astrocytes in the LC was associated with MERTK, we co-labeled GFAP and MERTK with AlexaFluor 488 and 594 in the LC. As expected, the co-location of GFAP and MERTK in the LC was significantly enhanced in the TBI group (Figure 7).

**Figure 7.**
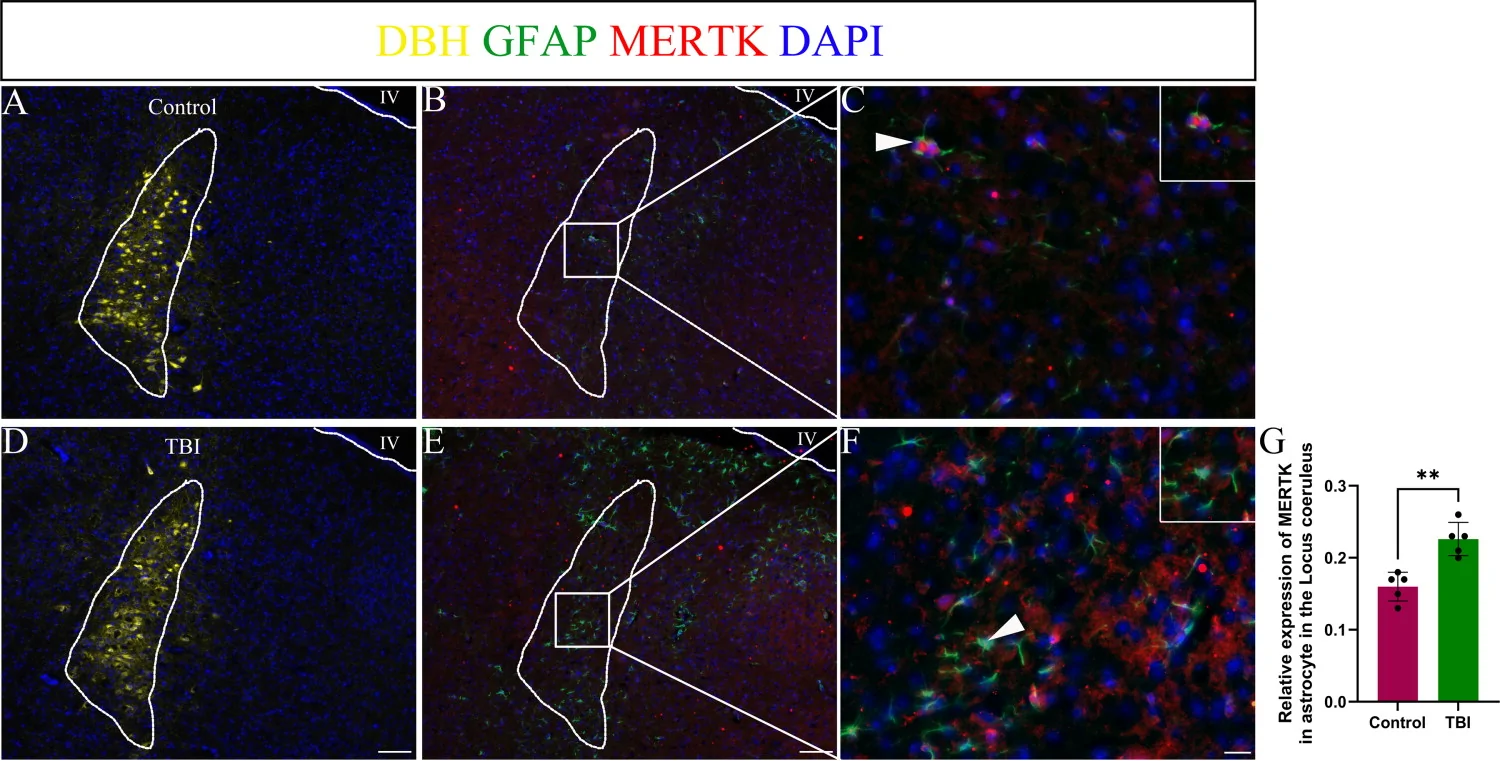
The expression of MERTK in astrocytes in LC increases following acute TBI. (A and D) The location of LC in control group and TBI group. The irregular dashed line outlining the range of DBH represents the LC. Scale bar: 100 μm. (B and C) Co-labeling of astrocytes and MERTK in LC of control group. Co-labeling of GFAP and MERTK in LC of control group were shown by the head of white arrows in (C), with the inset in the upper right corner showing an enlarged view of the indicated area. (E and F) Co-labeling of astrocytes and MERTK in LC of TBI group. Co-labeling of GFAP and MERTK in LC of TBI group were shown by the head of white arrows in (F), with the inset in the upper right corner showing an enlarged view of the indicated area. (B and E) Scale bar: 100 μm. (C and F)Scale bar: 20 μm. (G) The expression of MERTK in astrocytes in LC increases following TBI (each group, *n* = 5). t-tests employed for (G). (Contol group vs. TBI group, **p = 0.001). IV:Fourth ventricle.

### 3.4. Effects of PNM on LC during the acute phase of TBI

We further analyzed the effects of PNM on LC of mice following acute TBI. The results showed that during the acute phase of TBI, PNM could inhibit the apoptosis of norepinephrinergic neurons in the LC (Supplementary Figure 1B-E). However, compared with the control group, the apoptosis of norepinephrinergic neurons in the LC remained significantly increased in mice that received PNM following acute TBI (Supplementary Figure 1A, C-E). Notably, following acute TBI, LF-PNM could significantly activate norepinephrinergic neurons in the LC, while HF-PNM showed less activation of these neurons (Supplementary Figure 2).

To clarify whether PNM would affect the phagocytic activity of astrocytes in the LC following acute TBI, we also conducted a series of immunofluorescence colocalization experiments. The results indicated that following acute TBI, LF-PNM further promotes reactive activation of astrocytes, whereas HF-PNM exerts no significant effect on astrocyte activation within the LC (Supplementary Figure 3). Besides, LF-PNM could further enhance the phagocyte activity of astrocytes in the LC, while HF-PNM would attenuate this phagocytic activity (Supplementary Figure 4 and 5). Compared to the TBI group, the co-labeled GFAP and MERTK in the LC showed a significantly increase in the TBI+LF-PNM group, while it was significantly decreased in the TBI+HF-PNM group (Supplementary Figure 6D-M). Compared with the control group, the co-labeling of GFAP and MERTK in the LC was significantly increased in the TBI group, the TBI+LF-PNM group, and the TBI+HF-PNM group (Supplementary Figure 6).

### 3.5. PNM improve related neurological functions during the acute phase of TBI

To evaluate the arousal and stress responses in mice with acute TBI, we performed two evaluations: righting reflex and tail-flick test. The results showed that mice in the TBI group took significantly longer to turn themselves over and needed extended reaction times in the tail-flick test compared to the control group. After acute TBI, LF-PNM significantly shortened the time required for mice to turn over and the tail-flick latency. During the acute phase of TBI, HF-PNM could significantly shortened the tail-flick latency, but could not shorten the time required for them to turn over (Figure 8A and B).

**Figure 8.**
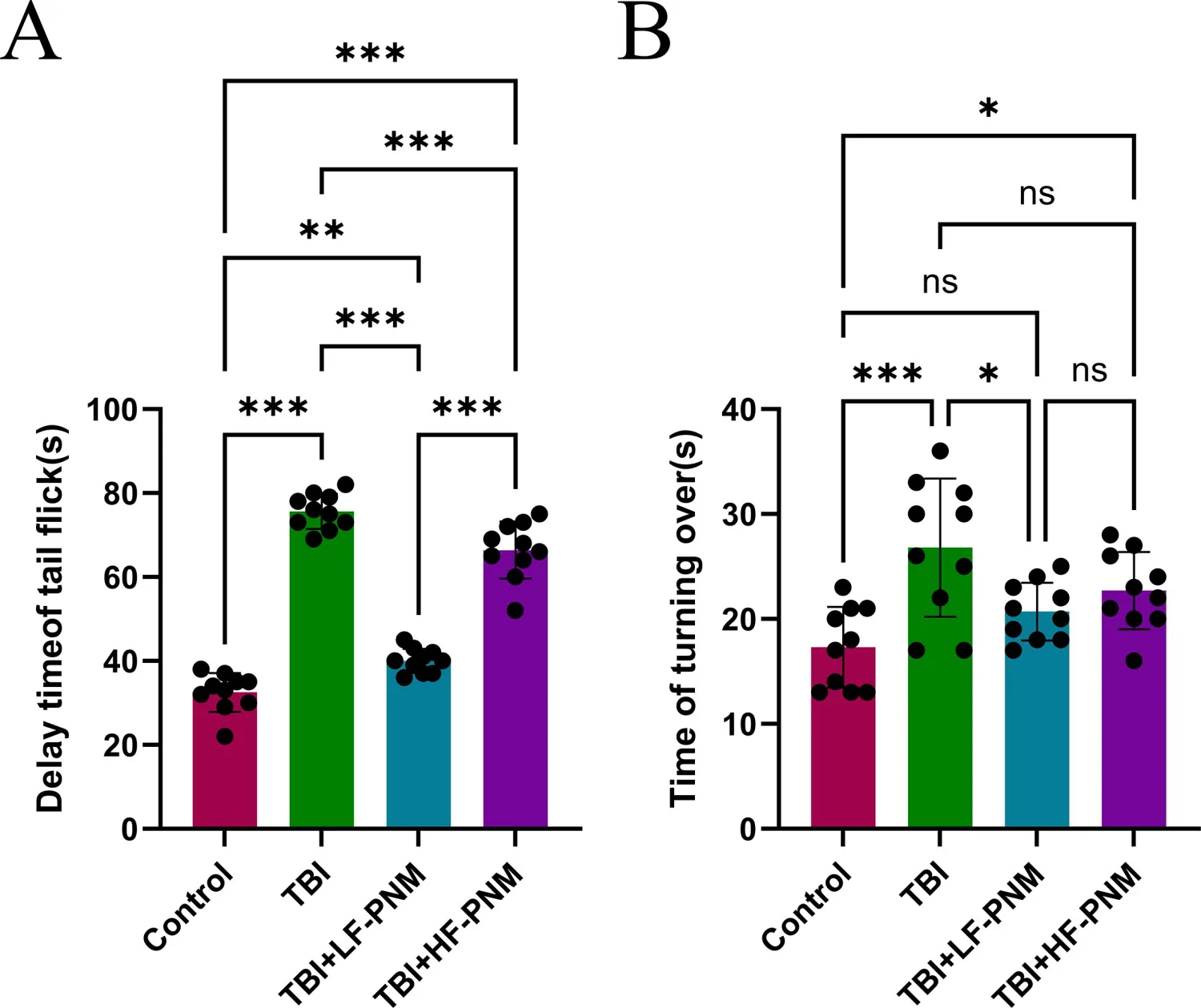
PNM improve related neurological functions during the acute phase of TBI. (A) The stress ability was evaluated using a tail-flick test, in which prolonged delay time represents poor stress ability. (Control group: 32.50 ± 4.65s; TBI group: 75.60 ± 4.17s; TBI+LF-PNM group: 40.00 ± 2.87s; TBI+HF-PNM group:66.40 ± 6.79s. Control group vs. TBI group, Control group vs. TBI+HF-PNM group, TBI group vs. TBI+LF-PNM group, TBI+LF-PNM group vs. TBI+HF-PNM group, ***p < 0.001; Control group vs. TBI+LF-PNM group, **p = 0.007; TBI group vs. TBI+HF-PNM group, ***p = 0.001). (B) The arousal ability was evaluated using a righting reflex test, in which prolonged turning over time represents poor arousal ability. (Control group: 17.30 ± 3.86s; TBI group: 26.80 ± 6.58s; TBI+LF-PNM group: 20.70 ± 2.75s; TBI+HF-PNM group:22.70 ± 3.68s. Control group vs. TBI group, ***p < 0.001; Control group vs. TBI+LF-PNM group, *p* = 0.335; Control group vs. TBI+HF-PNM group, *p = 0.048; TBI group vs. TBI+LF-PNM group, *p = 0.021; TBI group vs. TBI+HF-PNM group, *p* = 0.186; TBI+LF-PNM group vs. TBI+HF-PNM group, *p* = 0.748).

Our results indicated that acute TBI impaired the arousal and stress ability of mice, while PNM could improve the arousal and stress ability, with LF-PNM being more effective.

## 4. Discussion

The current study mainly found that: (1) During the acute phase of TBI, a large number of norepinephrinergic neurons in the LC underwent apoptosis, while astrocytes exhibited prominent reactive activation. (2) Following acute TBI, the phagocyte of astrocytes in the LC of mice was significantly enhanced. These astrocytes engulfed a large number of presynaptic membranes of norepinephrinergic neuronal axon terminals through the MERTK-related pathway. (3) PNM could inhibit the apoptosis of norepinephrinergic neurons in the LC after acute TBI. Particularly, LF-PNM could activate a large number of norepinephrinergic neurons, while HF-PNM had no significant effect on the activation of these neurons. During the acute phase of TBI, LF-PNM could further promote reactive activation and enhanced the phagocytic activity of astrocytes in the LC. By contrast, HF-PNM had no significant effect on astrocyte activation within the LC but attenuated their phagocytic capacity. (4) Acute TBI could lead to dysfunction in arousal and stress response. PNM could improve the related neurological functions, with LF-PNM being more effective.

### 4.1. Causes of arousal and stress dysfunction after acute TBI

The wide variability in disorders of arousal induced by TBI is mainly due to damage to the reticular formation in the brainstem [12]. The locus coeruleus, as a key nucleus in the reticular formation that serves as a bridge between higher and lower brain structures, is particularly vulnerable to damage [35]. Under normal conditions, the norepinephrinergic neurons in the LC project fibers that connect with the basal forebrain to regulate the arousal function [36,37]. Following acute TBI, apoptosis of norepinephrinergic neurons in the LC and disruption of neural circuits can lead to dysfunction of arousal. Arousal and stress are two interrelated and interdependent processes [38]. Therefore, damage to the LC after TBI could affects the arousal function as well as the stress function. In our study, the increased apoptosis of norepinephrinergic neurons in the LC after acute TBI in mice was the main factor leading to disorders of arousal and stress function.

### 4.2. Beneficial role of astrocytic phagocytosis in the LC after acute TBI

Numerous studies have shown that damage to the connections between LC-related pathways can lead to disruption of the arousal and stress processes [39,40]. These neural circuits are composed of neurons, axons, synapses, and supporting cells [41]. Glial cells stabilize these circuits by removing redundant synapses, promoting synaptic development, and assisting in the transmission of neurotransmitters between synapses [42]. A study by Anna Erlandsson confirmed that astrocytes actively collected and engulfed whole dead cells in an *in vitro* model of brain injury and thereby protected healthy neurons from bystander cell death [43]. In our study, following acute TBI, a large number of norepinephrinergic neurons in the LC underwent apoptosis, and the neural circuits related to LC may be disrupted. To clear redundant synapses and maintain the stability of neural circuits, the phagocyte of astrocytes in the LC was enhanced. Therefore, our study believed that the phagocytic activity of astrocytes on the synaptic terminals of norepinephrinergic neurons in the LC after acute TBI was beneficial.

### 4.3. Mechanisms of astrocytic phagocytosis in the LC following acute TBI

To further investigate the mechanisms of astrocytic phagocytosis of norepinephrinergic neurons in the LC of mice after acute TBI, we conducted Cellchat analysis and immunofluorescence colocalization. Historically, microglia have been considered as the only cells responsible for phagocytosis in the central nervous system [44]. After brain injury, their ability to swallow and digest is very important for clearing damaged neurons, nerve fibers and synaptic connections in the first stage of trauma [45]. In addition, many studies show that complementary systems (C3 and C1q) and interleukin-13 are responsible for controlling the phagocytosis of microglia after traumatic brain injury [46,47]. Interestingly, our CellChat analysis suggested that in the brainstem, astrocytes were more likely to recognize and phagocytose norepinephrinergic neurons compared to microglia. Studies have shown that astrocytes have many genes related to inflammation, such as MERTK and Megf10 [22]. Zhou et al. demonstrated that after TBI, the AXL/STAT1/ABCA1 signaling pathway mediated the transformation of astrocytes into a phagocytic phenotype [34]. This process effectively separated healthy brain tissue from cell debris caused by injury, promoted efficient phagocytosis of cell debris by astrocytes, and further alleviated neuroinflammation and neurological dysfunction following TBI. Similarly, after acute TBI, we observed prominent reactive activation of astrocytes within the LC. Moreover, the phagocytic activity of astrocytes on the presynaptic membranes of norepinephrinergic neurons was enhanced, and the expression level of MERTK in astrocytes increased.

### 4.4. Effects of PNM on astrocytic phagocytosis in the LC and neural function after acute TBI

Phagocytosis is a very important mechanism to keep the balance of neural circuits. It actively removes waste from cells, such as dead cells, synaptic elements and other debris. This process is particularly important in the formation of nervous system, because it helps to adjust synaptic connections and make the network well organized. The selective elimination of unnecessary synapses by phagocytosis will eventually help to enhance the stability of the established neural circuits [48]. Similarly, astrocytes in the LC remove synapses during acute traumatic brain injury. Mertk promotes this process, which helps to maintain the stability of neural circuits [22]. Throughout lifespan, microglia continue to devour dying progenitor cells. However, in the case of brain injury, this eating process is used to prevent the discharge of harmful substances, and eliminating the damaged medulla is very important to help axon regeneration. We observed that following acute TBI, LF-PNM in mice improved arousal and stress dysfunction, and the phagocytosis of presynaptic membrane proteins of norepinephrinergic neurons by astrocytes in the LC was particularly pronounced. Although HF-PNM after acute TBI could inhibit apoptosis of norepinephrinergic neurons in the LC, it also suppressed the phagocytic capacity of astrocytes. Therefore, HF-PNM after acute TBI was less effective in improving arousal and stress functions than LF-PNM.

### 4.5. Limitations

Our study had several limitations. We found that there was a positive correlation between the phagocytic activity of astrocytes in the LC and the upregulation of MERTK expression within these cells. We tentatively speculated that this may promote the phagocytosis of presynaptic membrane proteins of norepinephrinergic neurons following acute TBI. However, to verify this conclusion, further research using MERTK knockout mouse models is still required. Besides, we found that LF-PNM and HF-PNM had significantly different effects on arousal and stress function in mice following acute TBI. Therefore, the frequency of PNM may be related to the improvement of neurological function in mice with acute TBI. Subsequent experiments will involve a frequency gradient to further verify the effects of different frequencies of PNM on arousal and stress function in mice with acute TBI, in order to identify the optimal frequency of PNM. Last but not least, GFAP is only labels a subset of astrocytes and additional markers can provide a deeper and more complex understanding of astrocyte function in TBI.

## 5. Conclusion

During the acute phase of TBI, a large number of norepinephrinergic neurons in the LC underwent apoptosis. Astrocytes phagocytosed presynaptic membrane proteins of norepinephrinergic neurons through the MERTK-related pathway, and compared to HF-PNM, LF-PNM could further enhance this phagocytic activity, thereby improving arousal and stress dysfunction in mice following acute TBI.

## Supplementary Material

Supplemental Material

## Data Availability

The datasets generated and/or analysed during the current study are not publicly available due to the need for further research use but are available from the corresponding author on reasonable request.

## A priori protocol statement

A written study protocol, including the research question, key design features (sample size: *n* = 60; block randomization with allocation concealment; blinded outcome assessment; humane endpoints: body weight loss >20% or BCS ≤2), and a pre-specified statistical analysis plan was prepared before the initiation of the study (December 11, 2024). The study protocol was not registered in a public repository. No deviations from the pre-specified analysis plan occurred.

## Health and safety statement

All experimental procedures described in this study were strictly performed in full compliance with the mandatory laboratory health and safety regulations and standard operating procedures of the institution. All potential biological and experimental hazards involved in the experimental operations, material applications and relevant protocols have been fully assessed, and appropriate safety warnings and standardized preventive precautions were strictly implemented throughout the entire experimental process to guarantee laboratory operational safety and personnel safety.

For all animal experiments involved in this research, the experimental design, animal feeding, behavioral testing and subsequent treatment procedures were conducted in accordance with the Consensus Author Guidelines on Animal Ethics and Welfare formulated by the International Association of Veterinary Editors and the standard guidelines for animal treatment in behavioral research and teaching. No unapproved experimental products, drugs or reagents for the described research purposes were used in this study, and all adopted materials and experimental protocols complied with internationally recognized academic and laboratory safety codes of practice.

## Statement

We confirm that the study is reported in accordance with ARRIVE guidelines.
