# Supplementary material for: MERTK-related engulfment of norepinephrinergic neuronal presynaptic membrane by astrocytes following acute TBI and potentiation effect of peripheral nerve modulation
Source: Ann Med. 2026 Sep 27;58(1):2735720. doi: 10.1080/07853890.2026.2735720 (PMC13629777; doi:10.1080/07853890.2026.2735720)
Supplement: Supplementary file 1 [file IANN_A_2735720_SM1186.zip › suppl_data/supplementary figure legends.docx]

**Supplementary Figure 1** PNM can reduce apoptosis of norepinephrinergic neurons in LC following acute TBI. (A) Co-labeling of norepinephrinergic neurons and caspase3 in LC of control group. Co-labeled DBH and caspase3 in LC of control group were shown by the head of white arrows in (A), with the inset in the lower left corner showing an enlarged view of the indicated area. (B) Co-labeling of norepinephrinergic neurons and caspase3 in LC of TBI group. Co-labeled DBH and caspase3 in LC of TBI group were shown by the head of white arrows in (B), with the inset in the lower left corner showing an enlarged view of the indicated area. (C) Co-labeling of norepinephrinergic neurons and caspase3 in LC of TBI+LF-PNM group. Co-labeled DBH and caspase3 in LC of TBI+LF-PNM group were shown by the head of white arrows in (C), with the inset in the lower left corner showing an enlarged view of the indicated area. (D) Co-labeling of norepinephrinergic neurons and caspase3 in LC of TBI+HF-PNM group. Co-labeled DBH and caspase3 in LC of TBI+HF-PNM group were shown by the head of white arrows in (D), with the inset in the lower left corner showing an enlarged view of the indicated area. (A, B, C and D) Sacle bar:100μm. (E) Percentage of caspase3^+^ cells within DBH^+^ neurons in the LC of Control group mice, TBI group mice, TBI+LF-PNM group mice and TBI+HF-PNM group mice. (each group, n=5). One-way ANOVA employed for (E). (Contol group vs. TBI group, TBI group vs. TBI+LF-PNM group, TBI group vs. TBI+HF-PNM group, ^***^p<0.001; Control group vs. TBI+LF-PNM group, ^**^p=0.001; Control group vs. TBI+HF-PNM group, ^***^p=0.001; TBI+LF-PNM group vs. TBI+HF-PNM group, p=0.999). IV:Fourth ventricle.

**Supplementary Figure 2** LF-PNM following acute TBI can activate norepinephrinergic neurons in LC.(A and B) Co-labeling of norepinephrinergic neurons and c-Fos in LC of control group. Co-labeled DBH and c-Fos in LC of control group were shown by the head of white arrows in (B). (C and D) Co-labeling of norepinephrinergic neurons and c-Fos in LC of TBI group. Co-labeled DBH and c-Fos in LC of TBI group were shown by the head of white arrows in (D). (E and F) Co-labeling of norepinephrinergic neurons and c-Fos in LC of TBI+LF-PNM group. Co-labeled DBH and c-Fos in LC of TBI+LF-PNM group were shown by the head of white arrows in (F). (G and H) Co-labeling of norepinephrinergic neurons and c-Fos in LC of TBI+HF-PNM group. Co-labeled DBH and c-Fos in LC of TBI+HF-PNM group were shown by the head of white arrows in (H). (A, C, E, G) Scale bar: 100μm. (B, D, F, H) Scale bar: 20μm. (I) Percentage of c-Fos^+^ cells within DBH^+^ neurons in the LC of Control group mice, TBI group mice, TBI+LF-PNM group mice and TBI+HF-PNM group mice. (each group, n=5). One-way ANOVA employed for (I). (Contol group vs. TBI+LF-PNM group, TBI group vs. TBI+LF-PNM group, TBI+HF-PNM group vs. TBI+LF-PNM group, ^***^p<0.001; Control group vs. TBI group, p=0.669; Control group vs. TBI+HF-PNM group, p=0.386; TBI group vs. TBI+HF-PNM group, p=0.959). IV:Fourth ventricle.

**Supplementary Figure 3** Following acute TBI, LF‑PNM further promotes the reactive activation of astrocytes in LC. (A and B) GFAP expression level in LC of the control group. GFAP in LC of control group were shown by the head of white arrows in (B). (C and D) GFAP expression level in LC of the TBI group. GFAP in LC of TBI group were shown by the head of white arrows in (D). (E and F) GFAP expression level in LC of the TBI+LF-PNM group. GFAP in LC of TBI+LF-PNM group were shown by the head of white arrows in (F). (G and H) GFAP expression level in LC of the TBI+HF-PNM group. GFAP in LC of TBI+HF-PNM group were shown by the head of white arrows in (H). (A, C, E, G) Scale bar: 100μm. (B, D, F, H) Scale bar: 20μm. (I) The number of astrocytes in LC of Control group mice, TBI group mice, TBI+LF-PNM group mice and TBI+HF-PNM group mice. (each group, n=5). One-way ANOVA employed for (I). (Control group vs. TBI group, Control group vs. TBI+LF-PNM group, Control group vs. TBI+HF-PNM group, TBI group vs. TBI+LF-PNM group, ^***^p<0.001; TBI group vs. TBI+HF-PNM group, p=0.106; TBI+LF-PNM group vs. TBI+HF-PNM group, ^**^p=0.003). IV:Fourth ventricle.

**Supplementary Figure 4** LF-PNM can further enhance the robust engulfment of NET by astrocytes in LC following acute TBI. (A and B) Co-labeling of astrocytes and NET in LC of control group. Co-labeling of GFAP and NET in LC of control group were shown by the head of white arrows in (B), with the inset in the upper right corner showing an enlarged view of the indicated area. (C and D) Co-labeling of astrocytes and NET in LC of TBI group. Co-labeling of GFAP and NET in LC of TBI group were shown by the head of white arrows in (D), with the inset in the upper right corner showing an enlarged view of the indicated area. (E and F) Co-labeling of astrocytes and NET in LC of TBI+LF-PNM group. Co-labeling of GFAP and NET in LC of TBI+LF-PNM group were shown by the head of white arrows in (F), with the inset in the upper right corner showing an enlarged view of the indicated area. (G and H) Co-labeling of astrocytes and NET in LC of TBI+HF-PNM group. Co-labeling of GFAP and NET in LC of TBI+HF-PNM group were shown by the head of white arrows in (H), with the inset in the upper right corner showing an enlarged view of the indicated area. (A, C, E, G) Scale bar: 100μm. (B, D, F, H) Scale bar: 20μm. (I)The engulfment ability of NET in astrocytes was assessed by the co-labeled ratio of GFAP to NET. (each group, n=5). One-way ANOVA employed for (I). (Control group vs. TBI group, Control group vs. TBI+LF-PNM group, TBI group vs. TBI+LF-PNM group, TBI+LF-PNM group vs. TBI+HF-PNM group, ^***^p<0.001; Control group vs. TBI+HF-PNM group, ^***^p=0.001; TBI group vs. TBI+HF-PNM group, ^*^p=0.021). IV:Fourth ventricle.

**Supplementary Figure 5** LF-PNM can further enhance the phagocytic ability of astrocytes in LC following acute TBI. (A, D, G and J) The location of LC in four groups. The irregular dashed line outlining the range of DBH represents LC. Scale bar: 100μm. (B and C) Co-labeling of astrocytes and Lamp2 in LC of control group. Co-labeling of GFAP and Lamp2 in LC of control group were shown by the head of white arrows in (C), with the inset in the upper right corner showing an enlarged view of the indicated area. (E and F) Co-labeling of astrocytes and Lamp2 in LC of TBI group. Co-labeling of GFAP and Lamp2 in LC of TBI group were shown by the head of white arrows in (F), with the inset in the upper right corner showing an enlarged view of the indicated area.(H and I) Co-labeling of astrocytes and Lamp2 in LC of TBI+LF-PNM group. Co-labeling of GFAP and Lamp2 in LC of TBI+LF-PNM group were shown by the head of white arrows in (I), with the inset in the upper right corner showing an enlarged view of the indicated area. (K and L) Co-labeling of astrocytes and Lamp2 in LC of TBI+HF-PNM group. Co-labeling of GFAP and Lamp2 in LC of TBI+HF-PNM group were shown by the head of white arrows in (L), with the inset in the upper right corner showing an enlarged view of the indicated area. (B, E, H, K) Scale bar: 100μm. (C, F, I, L)Scale bar: 20μm. (M)Co-labeling of Lamp2 and GFAP in LC is enhanced following TBI, which indicates an increased phagocytic capacity of astrocytes. This phagocytic capacity can be further activated by LF-PNM. (each group, n=5). One-way ANOVA employed for (M). (Control group vs. TBI group, Control group vs. TBI+LF-PNM group, TBI group vs. TBI+LF-PNM group, TBI+LF-PNM group vs. TBI+HF-HF-PNM group, ^***^p<0.001; Control group vs. TBI+HF-PNM, ^**^p=0.003; TBI group vs. TBI+HF-PNM group, ^*^p=0.035). IV:Fourth ventricle.

**Supplementary Figure 6** LF-PNM can further increase the expression of MERTK in astrocytes in LC following acute TBI. (A, D, G and J) The location of LC in four groups. The irregular dashed line outlining the range of DBH represents LC. Scale bar: 100μm. (B and C) Co-labeling of astrocytes and MERTK in LC of the control group. Co-labeling of GFAP and MERTK in LC of control group were shown by the head of white arrows in (C), with the inset in the upper right corner showing an enlarged view of the indicated area. (E and F) Co-labeling of astrocytes and MERTK in LC of TBI group. Co-labeling of GFAP and MERTK in LC of TBI group were shown by the head of white arrows in (F), with the inset in the upper right corner showing an enlarged view of the indicated area. (H and I) Co-labeling of astrocytes and MERTK in LC of TBI+LF-PNM group. Co-labeling of GFAP and MERTK in LC of TBI+LF-PNM group were shown by the head of white arrows in (I), with the inset in the upper right corner showing an enlarged view of the indicated area. (K and L) Co-labeling of astrocytes and MERTK in LC of TBI+HF-PNM group. Co-labeling of GFAP and MERTK in LC of TBI+HF-PNM group were shown by the head of white arrows in (L), with the inset in the upper right corner showing an enlarged view of the indicated area. (B, E, H, K) Scale bar: 100μm. (C, F, I, L)Scale bar: 20μm. (M) The expression of MERTK in astrocytes in LC increases following TBI, which is further enhanced in astrocytes in LC after LF-PNM. (each group, n=5). One-way ANOVA employed for (M). (Control group vs. TBI group, Control group vs. TBI+LF-PNM group, TBI group vs. TBI+ LF-PNM group, TBI+LF-PNM group vs. TBI+HF-HF-PNM group, ^***^p<0.001; Control group vs. TBI+HF-PNM, ^***^p=0.001; TBI group vs. TBI+HF-PNM group, ^*^p=0.040). IV:Fourth ventricle.
